# Progress in Toxicology

**Published:** 1899-05-27

**Authors:** 


### PROGRESS IN TOXICOLOGY.

### FOOD POISONING.

Durham,[1] from an investigation of several outbreaks of food poisoning, concludes that the condition is an actual infection by living bacilli rather than an illness caused by ptomains, chemical substances easily destroyed by brief exposure to high temperature. Of the bacteria concerned the chief are Gärtner's Bacillus enteritidis, and Van Ermenghen's Bacillus botulinus. An outbreak of disease investigated by him took place in Chadderton (Oldham), 54 persons being attacked with gastro-intestinal symptoms after eating veal pies. There were four deaths. This outbreak he attributes to the B. enteritidis, and the blood of many sufferers gave a good reaction towards cultures of various varieties of this organism. Other food from the same shop gave rise to illness, which would tend to confirm the theory of a microbial origin. Probably one batch of pies was not heated to a temperature sufficient to destroy the bacillus. The organism was obtained from cultures of the meat. In cases that could be traced it is noteworthy that the animal which supplied the meat was itself diseased. The cow and calf figure most often in infections by B. enteritidis. B. botulinus has been associated with ham, and outbreaks of psittocosis in France have been attributed to Gärtner's bacillus. No cases have been connected with mutton. Milk may be infected by B. enteritidis. Fresh meat, if infected, is less likely to give rise to illness than meat that has been kept, as the bacilli have not had time to penetrate, and are easily destroyed in cooking. Exposure to sewer-gas cannot infect meat, nor are meats containing much gelatiniform material especially liable to contamination. In addition to the gastro-intestinal symptoms there may be pneumonia, rashes, herpes, desquamation, pain in the joints, limbs, and back. In investigating the cause of an outbreak all suspected articles should be seized, and kept in a refrigerator until cultures can be made. In case of death cultures should be made from the liver, kidney, spleen, and heart blood within 12 hours, or the organs wrapped in an antiseptic cloth, and sent at once for examination. Specimens of blood should be taken from sufferers for subsequent examination by serum diagnosis. Van Ermenghen[2] investigated an outbreak of poisoning affecting 20 persons, three of whom died within a week. The food partaken of was raw pickled ham, which, although slightly altered in taste, had no odour of putridity. No ptomains or other poisons could be detected. Other parts of the same pig gave rise to no symptoms. The symptoms were of an unusual character, and followed 24 hours after taking the food. There was pain, nausea, and vomiting of gelatinous black matter. Constipation was the rule in most cases. On the second day the sight became affected, amphodiplopia, dilatation of the pupils, loss of light reflex, and ptosis. Normal vision was not restored for six or eight months. The mucous membranes of the respiratory and digestive tracts were dry, and coated with grey mucous masses. Dysuria and anuria were common, and extreme muscular weakness was complained of. There was no rise of temperature. In fatal cases there was delirium, coma, and collapse. Cultures from the meat showed an anaërobic, spore-bearing bacillus, and the same organism was detected after death in the spleen of fatal cases. The bacillus has been named the B. botulinus, and the active toxin which it creates has been isolated by Brieger. This toxin is known as botuline, and is of such potency that one thousandth part of a millegram will kill a rabbit in 24 hours. It is destroyed by a temperature of 60-70 deg. C., and the bacillus itself dies at 85 deg. C., so that efficient cooking is sufficient to ensure safety.

Brieger and Kempner,[3] experimenting with a toxin obtained from Ermenghen's culture of B. botulinus, were able by administering this poison to animals to obtain a powerful antitoxic serum. They find the toxin to closely resemble that of diphtheria and tetanus, and to be formed in decomposing meat only when the specific bacillus is present. They do not consider that the B. coli is capable of exciting meat poisoning, but streptococcus may do so. Infected into animals botulinus toxin induces a fatal cachexia, with paralysis, and degenerative changes in spleen, liver, and kidneys.

Klein's[4] report on the Bacillus enteritidis sporogenes and its association with infantile diarrhœa and cholera nostras is of great interest. Certain infantile diarrhœa outbreaks may be traced to toxic bodies formed in the milk by spore-bearing bacilli, such as those described by Zübbert, or by the invasion of virulent streptococci (Escherich); but the Bacillus enteritidis sporogenes is the most common organism associated with summer diarrhœa, although absent from normal fæces or the evacuations of other diarrhœic diseases. The bacilli are cylindrical, 1·6 to 4·8m. in length and ·8m. in breadth. In serum cultures and in subcutaneous fluid spores are formed at one end of the rod. The organism is an obligatory anaërobe, grows on blood serum and gelatine with liquefaction, and forms gas in glucose agar. Its most characteristic reaction is seen when grown anaërobically in milk, viz., rapid and copious gas formation, separation of the milk into casein flocculi, and into clear thin watery whey smelling of butyric acid and giving an acid reaction. Injected into rabbits death results in 24 hours, with extensive subcutaneous effusion, and sanguineous exudation into the intestines and peritoneum. The bacilli are found in all the organs. In typical summer diarrhœa this bacillus is constantly present, and Klein isolated its spores in four out of eleven cases, first heating to 80 deg. C. for ten minutes, and then growing in anaërobic milk cultures. Six out of eight cases of cholera nostras gave positive results, and of ten samples of milk tested eight contained the Bacillus enteritidis, which was also found in one out of three samples of sterilised milk. It is also present in sewage, in horse manure, and in manured soils.

**Tinned Foods.**—The numerous fatal cases of poisoning by tinned food recorded during the past twelve months and the large seizures in London of decomposing tinned materials have drawn considerable attention to this form of food poisoning. There are three possible causes of poisoning. It may be due to metallic substances obtained from the tins or the solder used in closing the tins; it may arise from direct microbial infection, owing to the food being imperfectly sterilised in course of preparation; or it may be due to the presence of ptomains formed by bacteria which were themselves destroyed in subsequent stages of the preparation. The methods of detecting tins containing decomposed materials and the measures required to check the importation of such have been discussed by Brown[5] and Alexander.[6] Decomposition of the meat is accompanied by the formation of gas which expands the ends of the tins, making them convex instead of concave. Such tins are spoken of as "blown" or "springy," and it is the custom amongst fraudulent dealers to prick the lid or side, allow the gas to escape, and then, after heating, to once more solder up the hole. In this way a tin may to all appearances be sound whilst the contents are of a highly poisonous nature. It is suggested that no can shall be sold which has more than one soldering point; that the original solder point shall be left uncovered by a label, and have stamped over it the name of the manufacturer and the date of manufacture. To prevent the stripping off of old labels, and puncturing at the side where a new label can be affixed, it is recommended that every label shall bear the name of the importer, who shall be responsible for the contents. Manufacturers are recommended to place their tins in hot rooms for several weeks to see if they "spring." To prevent metallic poison, terne-plated cans, that is, iron cans coated with an alloy of lead and tin, shall not be used, especially for fruit. Alexander mentions that properly-sealed canned food will keep for 30 years and longer, some manufacturers having still tinned provisions sound and intact which were prepared for use in the Crimean campaign.

The addition of colouring materials containing copper salts to food is a not uncommon practice. Copper salts are chiefly employed to give a bright green colour to preserved vegetables and fruits, such as peas and greengages. It would, however, seem certain that such foods, if used frequently, are capable of exciting chronic poisoning.[7] The most dangerous salts are the oleate, acetate, and sulphate. Symptoms manifest themselves in about the fourth week, and may not disappear for three or four months. Copper is not eliminated in the milk, but has been found to enter the fœtal circulation.

[1] Brit. Med. Jour., Dec. 17, 1898. [2] Zeitsch. f. Hygiene und Infect., xxvi., p. 1. [3] Deut. Med. Woch., Aug. 12, 1897. [4] Medical Officer's Suppl., Local Government Board, 1897-98. [5] Jour. of Sanit. Instit., 1898. [6] Lancet, May 13, 1899. [7] Zeit. öff Chem., 1898.

